# Spirituality and Psychological Well-Being of Adults with a History of Child Abuse by Catholic Clergy: A Systematic Review of Qualitative and Quantitative Studies

**DOI:** 10.1007/s10943-025-02379-3

**Published:** 2025-07-01

**Authors:** Dhanach Dhirachaikulpanich, Wichapol Dendumrongsup, Thamonwan Viyoch, Nattacha Srithawatpong, Thanakorn Angkasirisan, Xi Huang, Sorawit Wainipitapong

**Affiliations:** 1 Faculty of Medicine Siriraj Hospital, Mahidol University, Bangkok, Thailand; 2 Faculty of Medicine, Chulalongkorn University and King Chulalongkorn Memorial, Hospital, Bangkok, Thailand; 3 Department of Experimental Psychology, University of Oxford, Oxford, UK; 4 Faculty of Education, Department of Educational Psychology, Chinese University of Hong, Kong, Hong Kong, China; 5 Faculty of Medicine, Department of Psychiatry and Center of Excellence in Transgender Health (CETH), Chulalongkorn University and King Chulalongkorn Memorial Hospital, Bangkok, Thailand; 6 Department of Global Health and Social Medicine, King’s College London, London, UK

**Keywords:** Psychological, Well-being, Spiritual, Abuse, Catholic, Christianity

## Abstract

This systematic review explored the spirituality and psychological well-being of adults with a history of child abuse by Catholic clergy. The study was registered with PROSPERO (CRD42023468440) and searched on MEDLINE, Embase, and PsycINFO. Five qualitative and eight quantitative studies were included. The studies reported varied psychological adversities. We developed three themes using thematic synthesis: 1) comprehensive yet suspicious self-negativity, 2) challenges in interacting with others, and 3) alterations in practices and beliefs in God and the Church. Despite the findings, causality and sociocultural factors remain underexplored, necessitating further studies for a deeper understanding and the development of supportive policies.

**Supplementary Information:**

The online version contains supplementary material available at 10.1007/s10943-025-02379-3.

## Introduction

Child abuse, including physical, emotional, and sexual harm to children, can occur in various settings, including homes, schools, communities, as well as religious institutions. This issue remains a pressing concern in research and clinical practices due to its profound and multifaceted impacts, extending from individual well-being to implications for societal and policy to prevent and mitigate child abuse (Bright et al., 2022). Numerous studies have investigated mental health outcomes of child abuse during adulthood, including drug misuse, suicides, behavioral problems, and various mental disorders (Angelakis et al., 2020; Norman et al., 2012). However, less attention has focused on how child abuse might affect spirituality, which is another important domain of many individuals’ well-being. This is a major concern especially for instances of child abuse that involved a spiritual or religious figure.

Specifically, within the context of the Catholic Church, which has stood her position as a pillar of moral and spiritual guidance for over a billion believers worldwide (Segreteria di Stato del Vaticano, 2019). The clergy, entrusted with roles central to the mission of the Church, have been revered as resources of trust, faith, and charity. However, child abuse cases by clergy members have shaken the very foundations of these values for the Catholic community, particularly for survivors of such abuse.

Few studies have explored the relationship between child abuse by Catholic clergy and spiritual consequences on survivors and families. These include a broad spectrum of mental disorders, such as anxiety, depression, and trauma-related disorders, mental health service utilization, and reported negative emotions (Dressing et al., 2017). For spirituality, changes in religious practices and attitudes toward the Church have been reported (McLaughlin, 1994).

Despite the past investigations on the psychological and spiritual outcomes of the survivors of child abuse by Catholic clergy, to our knowledge, no systematic review has been conducted to explore the psychological well-being and spirituality of adults with a history of child abuse by Catholic clergy. This limits researchers and policymakers to systematically and accurately infer about the consequences of child abuse by Catholic clergy, which are essential for the Catholic Church and other religious institutions in shaping potential policies aimed at protection, healing, and accountability (Hailes et al., 2019). Our review, therefore, aims to systematically explore the psychological well-being and spirituality of adults with a history of child abuse by Catholic clergy.

## Materials and Methods

### Search Strategy

This review was registered with PROSPERO (CRD42023468440) and adhered the PRISMA guidelines (Moher et al., 2009). We conducted a narrative synthesis to summarize our findings. Ethics approval was not required. In November 2023, three databases (MEDLINE, Embase, and PsycINFO) were systematically searched using comprehensive search terms related to: 1) psychological or spiritual, 2) abuse, and 3) Catholic clergy (Appendix 1). These search terms were combined using the “AND” operator across all three domains. Searched encompassed the abstract, title, and keyword fields. Furthermore, we manually searched relevant reviews and references to identify additional sources.

### Eligibility

We included studies examining the psychological well-being or mental status and spirituality of adults with a history of childhood abuse by Catholic clergies, published in peer-reviewed journals, without restrictions on publication date. Exclusion criteria comprised: 1) publications not in English, 2) insufficient data or unpublished sources, and 3) any forms of reviews and meta-analyses.

### Study Selection, Data Extraction, and Quality Appraisal

WD handled deduplication of search results and, alongside KT, independently conducted titles and abstracts screening. SW and DD performed the full-text assessments, and data extraction was carried out by DD. Any disagreements or uncertainties during the review and data extraction process were resolved through panel discussions involving all authors. DD assessed the methodological quality of included studies using the JBI instruments (Barker et al., 2023).

Finally, SW randomly reassessed 10% of extracted data and quality appraisal.

### Data Synthesis

Data from included quantitative studies were integrated in a narrative synthesis according to examined outcomes. Statistical values such as count, percentage, mean, and standard deviation were extracted/calculated and tabulated.

For the qualitative data synthesis, we employed the thematic synthesis methods using inductive coding (Thomas & Harden, 2008). The initial line by-line coding was conducted by SW, utilizing the results and discussion sections of all included papers. DD then apply these codes to the included studies, suggesting new codes if necessary. Subsequently, the codes will be discussed, agreed upon, and either grouped or collapsed, forming descriptive themes. Finally, analytic themes will be developed using the map of descriptive themes and codes.

## Results

**Fig. 1:**
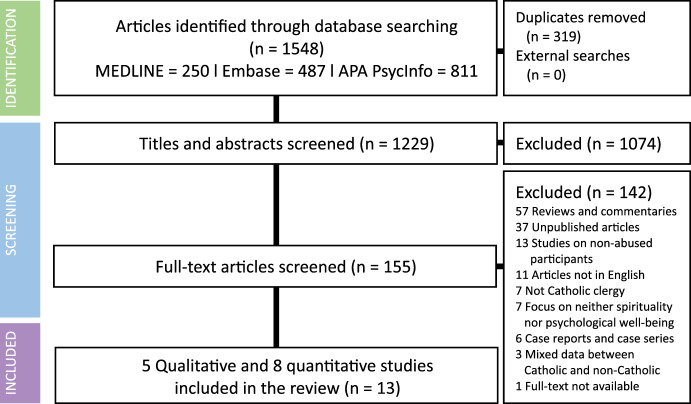
PRISMA flow diagram

Thirteen studies were eligible for inclusion (Fig. 1), comprising five qualitative studies and eight quantitative studies, of which seven and one were cross-sectional and descriptive studies reviewing documents from clergy, respectively. The majority of studies were conducted in the USA (n = 5; 38.5%) and predominantly focused on sexual abuse. Of all studies, each three focused solely on spirituality and psychological well-being, and the remaining seven investigated both outcomes. A summary of general and methodological profiles of all included studies is presented in Table 1.

**Table 1:** General and methodological profiles of studies included

First author, year	Study location	Study design	Year of data collection	Method of recruitment	Types of abuse	Outcomes of interest	Comparison group for this review	Data analysis method
McLaughlin, 1994	USA	Cross-sectional	1994	Convenience sampling of participants in a conference	Sexual	Spirituality: M.O.S.T and QDS	Protestant survivors	Analytic statistics (χ^2^ and correlation coefficient)
Mart, 2004	*USA*	*Qualitative*	*N/A*	*Purposive sampling from alleged victims*	*N/A*	*Spirituality: Interview* *Psychological: PAI and DAPS*	*None*	*Narrative synthesis*
*Isely et al., *2008	*USA*	*Qualitative*	*2008*	*Purposive sampling from relevant network*	*Sexual*	*Spirituality and Psychological:* *Interview*	*None*	*Theme analysis*
Shea, 2008	USA	Cross-sectional	N/A	Voluntary sampling by sending email	Sexual	Spirituality: SCSORF and QDS Psychological: LHS, BDI, PCL-S, and QDS	Survivors abused by others not a priest	Analytic statistics (Independent *t-*test)
Farrell, 2009	*UK*	*Qualitative*	*2007*	*Voluntary sampling in relevant organization*	*Sexual*	*Spirituality: Interview; Psychological: PTSD-QAI, ITQ, and interview*	*None*	*IPA*
Lueger-Schuster, 2014a	Austria	Cross-sectional	2012	Voluntary sampling by letters and some gave permission for further contacts and research	Three types: physical, emotional, and sexual	Psychological: PCL-C, BSI, and GSI	None	Analytic statistics (χ^2^, *t*-test, and *U*-test)
Lueger-Schuster, 2014b	Austria	Cross-sectional	2012	Voluntary sampling by letters distributed by the commission	Three types: physical, emotional, and sexual	Psychological: PCL-C, BSI, CISS, DLE, CD-RISC, LOT, and RPSSQ	None	Analytic statistics (χ^2^ and ANOVA)
Spröber et al., 2014	Germany	Cross-sectional	2010–2011	Reporting system for victims of abuse	Mainly sexual, physical, and emotional	Psychological: Self-reported mental disorders and psychosocial problems	Protestant and non-religiously affiliated contexts	Analytic statistics (Kruskal–Wallis test and *U*-test)
Dreßing et al., 2019	Germany	Descriptive	1946–2014	Personnel records of clergy	Sexual	Spirituality and Psychological: Records from the documents	None	Only descriptive statistics
*Easton et al.,* 2019	*USA*	*Qualitative*	*2010*	*Purposive and voluntary sampling*	*Sexual*	*Spiritual and Psychological: Textual* *responses in open-ended question* *in the online survey*	*None*	*Conventional content analysis*
Pereda & Segura, 2021	Spain	Cross-sectional	2018–2019	Convenient and snowball sampling	Sexual	Spirituality: QDS	None	Only descriptive statistics
Pereda et al., 2022	Spain and Chile	Cross-sectional	N/A	Convenient sampling by advertisement of relevant organizations	Sexual	Spirituality: SIS and QDS Psychological: QDS (mental health and social problems)	Survivors abused by non-Church (within and without family)	Analytic statistics (χ^2^, Fisher’s exact test, ANOVA, and regressions)
*Prusak & Schab, *2022	*Poland*	*Qualitative*	*2018–2019*	*Convenient and snowball sampling*	*Sexual*	*Spirituality: Interview*	*None*	*IPA*

*M.O.S.T* Measure of Spirituality Test, *QDS* Questionnaires developed for the study, *PAI* Personality Assessment Inventor, *DAPS* Detailed Assessment of Post-traumatic Stress, *SCSORF* Santa Clara Strength of Religious Faith Scale, *LHS* Learned Helplessness Scale, *BDI* Beck Depression Inventory, *PCL-S* Post-traumatic Stress Disorder Checklist-Specific, *PTSD-QAI* post-traumatic stress disorder qualitative assessment, *ITQ* Idiosyncratic Trauma Questionnaire, *IPA* Interpretative phenomenological analysis, *PCL-C* PTSD Checklist-Civilian Version, *BSI* Brief Symptom Inventory, *CISS* Coping Inventory for Stressful Situations, *DLE* Disclosure of Loss Experience, *CD-RSIC* 10-item Connor–Davidson Resilience Scale, *LOT* Life Orientation Test, *RPSSQ* Recalled Perceived Social Support Questionnaire, *SIS* Spiritual Injury Scale

Table 2 outlines the findings from each qualitative and quantitative study included in this review. Sample size varied, ranging from 26 to 3677, and several measures assessing spirituality and psychological well-being were utilized. On average, participants were their 40 s and 50 s, and the onset of abuse occurred around the ages 10–13.

**Table 2:** Summary of findings of all included quantitative studies

First author, year	Sample size	Age (*M*)	AGE (*SD*)	%Male	Age at first abuse (*M*)	Results summation	Note
McLaughlin, 1994	26	58% aged between 40 and 49	N/A	48	Mostly children	65% reported therapy as a peace space; 27% mistrust of church/clergy and feeling of isolation; 58% did not attend church services; 14% changed religious affiliation; and 32% did not involve with any church after abuse	Retrospective scoring; all participants from the compared group (n = 8) were abused during adults
Shea, 2008	29	50.9	10.7	100.0	69% aged between 10 and 13	Mean of SCSORT = 21.31 (low faith), LHS = 46.8 (out of 80; no cutoff recommended), BDI = 20.8 (moderate depression), and PCL-S = 54.3 (possible PTSD), none reached statistical significance compared to controls; significantly lower scores of perceptions of and belief in church	Four of 29 abused by a religious brother; compared group (n = 20) were significantly younger with mean age of 43.3 years
Lueger-Schuster, 2014a	448 & 185	55.1 & 56.3	10.5 & 9.5	75.7 & 76.1	N/A & 10.0	48.6% screened positive for PTSD; 84.8% scored positive for at least one clinically psychopathological symptom	53.1% perpetrators were diocese priests or male monastics; PTSD screened in 185 participants
Lueger-Schuster, 2014a	185	56.3	9.5	76.1	10.0	15.1% no PTSD symptom & 56.8% high PTSD symptoms; Mean of CISS = 32.3 (out of 96), DLE = 30.2 (out of 60), CD-RSIC = 23.2 (out of 40), and RPSSQ = 13.7 (out of 40), cutoff of all measures was not provided	Focus on coping, loss experience, social supports, and resilience; three groups categorized by levels of PTSD symptoms
Spröber et al., 2014	404	54.9	13.0	69.8	N/A	80.2% reported at least one mental health disorder; diagnosis 45.0% depression, 17.4% PTSD, 14.7% anxiety; Psychosocial issues 21.6% health, 18.8% relationship and partnership, and 16.2% flashbacks/intrusion/nightmare	85.9% by male offenders 8.5% by female, and 5.6% by both; none reached statistical significance compared to other two groups
Dreßing et al., 2019	3677	42.6	11.3	62.8%	51.6% aged ≤ 13 years	17.0% received psychiatric treatment; social function problems 53.1% relationship, 43.0% sex life, 34.2% career, 32.5% social participation; health consequences 42.4% fears, 42.3% depression, 28.7% distrust, 9.3% suicide attempt, 4.9% self-harm; and 3.9% left the church	No valid clinical diagnosis and improper methodology of investigating outcomes; health consequences were stated in 1028 participants
Pereda & Segura, 2021	38	51.1	11.7	65.8%	11.8	Extreme or considerable impact on belief in church (68.5%) and in God (44.8%); 44.7% little or no impact on faith in God; and 7.9% moderate impact on belief in both	Self-reported from participants; 7.9% reported country of birth as not Spain
Pereda et al., 2022	40	48.5	11.8	72.5%	N/A	Impact on Beliefs in God 42.5% none or little, 45.0% considerable too extreme, reached statistical significance; 82.5% either mental or social problems (not significant)	Country of birth 67.5% Spain, 25.0% Chile, and 7.5% others

*SCSORT* Santa Clara Strength of Religious Faith Scale, *LHS* Learned Helplessness Scale, *BDI* Beck Depression Inventory, *PCL-S* – Post-traumatic Stress Disorder Checklist-Specific, *PTSD* Post-traumatic Stress Disorder, *CISS* Coping Inventory for Stressful Situations, *DLE* Disclosure of Loss Experience, *CD-RSIC* 10-item Connor–Davidson Resilience Scale, *RPSSQ* Recalled Perceived Social Support Questionnaire, ^a^ study on adult survivors of institutional abuse in settings connected to the Catholic Church, not clearly stated as by Catholic clergy; ^b^ study on abuse in Catholic contexts, not clearly stated as by Catholic Clergy

Summarized qualitative findings are presented in Table 3. One study focused on female participants, one did not provide gender details, and the remaining three explored male survivors. In terms of data collection and analysis, four studies utilized the interview approach with narrative synthesis, theme analysis, and interpretative phenomenology analysis. One study employed open-ended questions and conventional content analysis.

**Table 3:** Summary of findings of all included qualitative studies

First author, year	Sample size	Age	%Male	Age at first abuse	Outcomes and Themes identified	Note
Mart, 2004	25	23–53	100.0%	N/A	Trends toward avoidant traits, social withdrawal, shyness, and unassertiveness, leading to vulnerability for victimization, closeness to religious activities, and fear of disclosure; religious disengagement despite strong religiosity background	High proportion participants with active church activities such as altar boys; no quotation provided; themes identified related to post-abuse outcomes were grouped in our review;
Isely et al., 2008	9	31–67	100.0%	9–15 years old	Immediate and long-term psychological impacts such as fear, shame, anger, confusion, intrusion, mistrust, and amnesia; social and behavioral consequences, feeling estranged from others, avoid relationship with men, concerning about orientation and identity, and sexual promiscuity	Only post-abuse outcomes were selected and grouped in our review
Farrell, 2009	12	44 on average	N/A	N/A	Unique trauma symptomatology after abuse 1) theological conflict, 2) idiosyncratic silencing strategies, 3) spiritual identity, 4) political anger, 5) existentialism, and 6) re-traumatization by the church	66.7% became agnostic or atheistic, attributed directly from abuse; and 83.3% received psychological treatment
Easton, 2019	205	27–78 (M = 52.0)	100.0%	M = 11.3	Impacts on self-identity: 1) total self, 2) psychological self, 3) relational self, 4) gendered self, 5) aspirational self, and 6) spiritual self-least mentioned (6.3%)	Majority (47.8%) reported psychological self-issues, including mental health problems, self-harm, and low self-esteem
Prusak & Schab, 2022	5	27–30 (M = 28.6)	0.0%	M = 16.8	1) God image (diverse perceptions), 2) emotions towards God (anger and fear), 3) sense of guilt before God, and 4) distrust of the institutional church and clergy, leading to loss of spiritual security	Four of five experienced religious struggle; some perceived as they allowed, or even induced, the abuse

*M* Mean

### Quality Appraisal

According to the JBI, the overall quality of all included studies was considered not with excellent quality (see Appendix 2). All qualitative studies did not clearly address the influence of the researcher on the research and vice versa. Meanwhile, in six out of seven quantitative studies, there was an unclear mention of confounders and how to manage them. Other domains rated as “unclear,” with a range of two to six papers per each domain, included details of subjects and settings, as well as valid and reliable way of measuring exposure and outcomes. Due to the availability of JBI’s specific methodological quality checklist, one descriptive study underwent appraised by the JBI for prevalence study, and clarity was lacking regarding for the valid method used for the identification of the condition.

### Qualitative Data Synthesis

**Fig. 2:**
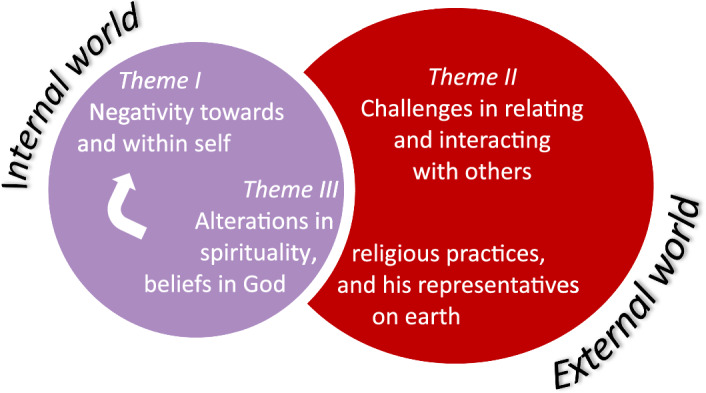
Main thematic findings

We identified three thematic clusters: 1) comprehensive yet suspicious negativity toward and within self, 2) challenges in relating and interacting with others, and 3) alterations in spirituality, religious practices, and beliefs in God and his representatives on earth. These themes reflect experiences both within the individual (internal world) and in interactions with others (external world), as illustrated in Fig. 2.

### Theme I—Comprehensive yet suspicious negativity toward and within self

Several negative emotions, including fear, shame, anger, obsession, intrusion, panic, hypervigilance, anxious, and confusion, occurred immediately after the abuse (Easton et al., 2019; Isely et al., 2008). Many of these emotions persisted into the long term and developed into a wide range of mental disorders, such as trauma-related disorder, depression, and self-harm (Easton et al., 2019; Farrell, 2009), despite the loss of memory of the abusive events in some survivors (Isely et al., 2008).

However, among various negative emotions that emerged, some survivors reported self-doubt, questioning whether they were the reason for the abuses (Prusak & Schab, 2022). This doubt was later transformed, internalized, or manifested as other negative emotions, including guilt. This doubt also extended beyond feelings to various aspects of self, such as underdevelopment and disconnectedness, doubting one’s orientations and masculinity, experiencing low self-esteem, and perceived loss of future and success (Easton et al., 2019).

“I somehow had it in my four-year old head that bad things happen to boys so if I was a girl, I would be safe. This progressed from fantasy and wishing to cross-dressing to, at one point, seriously considering... sexual reassignment” – Participant 290 (Easton et al., 2019).

Additionally, the vulnerable characteristics of participants suggested an avoidant and less assertive trait (Mart, 2004), as well as passivity (Easton et al., 2019). This trait could either be a post-abuse consequence or a pre-abuse predisposing factor that provided opportunities for abuse. Moreover, these characteristics prolonged actions seeking help due to their nature of reluctance, along with complex emotion aftermaths, as described above.

### Theme II—Challenges in relating and interacting with others

*“I mean, if you and I couldn’t trust our parish priest; *excuse* me.*

*Point out someone to me that I can.”* – Participant (Isely et al., 2008).

Pre-abuse trust in clergy was highly esteemed, not only by the survivors but also by their family members (Mart, 2004; Prusak & Schab, 2022). Some survivors used to perceive such clergy as a source of trust and love, akin to a father figure (Isely et al., 2008). However, the abusive events shattered this trust, leading to social and behavioral consequences in adulthood, including feelings of estrangement from others (Isely et al., 2008) or social isolation due to fear of stigma (Easton et al., 2019).

These consequences then manifested in difficulties with interpersonal relationships, particularly with men and authorities, as well as in intimate relationships. In some survivors, these unhealthy relationships subsequently led to sexual promiscuity and compulsive sexual activities (Easton et al., 2019; Isely et al., 2008).

*“I struggle with monogamy with *the* most wonderful person of 12 years,*

*and I cheated numerous times in *bathrooms*, cars, places on the go,*

*and it has led to a constant *unhappiness* in what started as a wonderful marriage.”*

– Participant 374 (Easton et al., 2019).

*Theme III—Alterations in spirituality, religious practices, and beliefs in God and his representatives on earth*

Many survivors discontinued their involvement in religious activities or changed their religious affiliations, despite their active participation in church activities (Easton et al., 2019; Farrell, 2009; Mart, 2004). Questions about God emerged, reflecting challenges to their belief in God and evoking diverse perceptions and emotions, including doubts about existence, reduced importance, guilt, ambivalence, and a perceived sense of disgust by God (Farrell, 2009; Prusak & Schab, 2022).

*“If God is meant to be so good, why didn’t He intervene and stop what was happening?*

*Why didn’t He help and protect me?”* – Survivor (Farrell, 2009)

The abuse had profound effects on survivors’ spirituality, extending beyond religious aspects to existential considerations, a sense of human existence, and contemplation of the afterlife (Farrell, 2009). Additionally, survivors reported alterations in their perceptions of the Catholic Church and clergy. Negative attitudes toward the Church were attributed to response actions perceived as unfair and re-traumatizing, resulting in anger and disappointment (Farrell, 2009; Prusak & Schab, 2022). Some survivors, therefore, chose to leave the Catholic Church while still maintaining their belief in God.

*“Priests, the clergy, the people in charge. Their ignorance, their pride.. you know,*

*It really gets me! I mean I can’t understand why *someone* like that is in charge. But of course, understanding the kind of mechanism of power, and understand or trying to understand what’s going on.. I guess.. I blame these people rather than God, right?”.*

– Joanna (Prusak & Schab, 2022).

## Discussion

Our review synthesized studies examining psychological status and spirituality during adulthood using quantitative and qualitative designs. Despite some papers claiming consequences of childhood abuse by Catholic clergy, none of the included studies employed longitudinal methodology. Therefore, as indicated in our title and throughout the manuscript, we avoided using the term “consequences” due to the nature of the included studies and their limitation for causal explanation.

Regarding psychological well-being, several negative psychological conditions were reported in a very wide range and direction, either within self or toward others. For direction toward others, mistrust was commonly examined and reported for approximately one-third of the survivor (Dreßing et al., 2019; McLaughlin, 1994). The alterations of trust was conceptualized as a pioneer and very foundation of further interactions as once trust is off, all bets are off (Porter-O’Grady, 2024). For example, psychosocial or relationship problems and social isolation were identified in our included studies (Dressing et al., 2017; Spröber et al., 2014).

Meanwhile, the poor well-being experienced by each survivor manifested in numerous psychiatric symptoms and sub-symptoms, including fears, anxiety, sexual issues, self-harm, suicidal attempts, alcohol misuse, and eating problems (Dreßing et al., 2019; Spröber et al., 2014). However, due to methodological limitations and a multitude of confounding factors, the generalizability of the prevalence reported in these studies should be approached with caution.

Interestingly, one US study compared these outcomes using a variety of questionnaires and found no significant differences between survivors of abuse by members of the Catholic Church and the Protestant (Shea, 2008). This may be attributed to the similar characteristics of these two groups of perpetrators as spiritual figures. However, two other studies from Germany, and from Spain and Chile, involving comparisons between the Catholic, Protestant, and non-religiously affiliated groups (Spröber et al., 2014) as well as between Church-affiliated and non-affiliated groups within or without familial abuse contexts (Pereda et al., 2022), also reported no statistical significance. These results highlighted the multifaceted factors at play and emphasize the need for further well-qualified studies in this area within different sociocultural backgrounds.

For mental disorders, most studies focused on conditions related to trauma, particularly post-traumatic stress disorder (PTSD). A meta-analysis of this condition was previously reported (Mcgraw et al., 2019), encompassing religious-affiliated perpetrators beyond the Catholic Church and regardless of peer-reviewed publication. However, the prevalence of PTSD identified in our review ranged between 17.4% and 48.6%, with each study utilizing different methods to assess PTSD. Nevertheless, this prevalence was still higher than that observed in the general population (3.9%) and individuals exposed to trauma (5.6%) (Koenen et al., 2017), underscoring the complexity and uniqueness of this type of abuse and its involvement with spirituality. The qualitative approaches, therefore, were crucially necessary to deeper explain this finding.

Beyond the single diagnosis of PTSD, three studies suggested the prevalence of other mental disorders. However, they used dissimilar approaches, with two reporting that 80.2% self-reported at least one mental illness (Spröber et al., 2014), and 84.8% had the presence of at least one psychopathological symptom (Lueger-Schuster et al., 2014a, 2014b), while the other reported 17.0% individuals receiving psychiatric treatment (Dreßing et al., 2019). This discrepancy can be attributed to methodological differences and restrictions, as mentioned earlier. Additionally, these outcomes did not necessarily align with the same definitions, as individuals may have a mental illness but avoid mental health services due to stigma (Chiddaycha & Wainipitapong, 2021). Therefore, it is imperative to provide a safe space for survivors to support their mental health. This was also emphasized by one study indicating that 65.0% of participants used therapy sessions as their peace spaces (McLaughlin, 1994), potentially after the Catholic Church lost her position as a safe and peaceful space for survivors and their spirituality.

Our reviews revealed that spirituality was examined using a variety of methodologies. While some studies employed questionnaires with binary responses or ordinal scales specifically developed for their research, three studies utilized the established measures of spirituality (McLaughlin, 1994; Pereda et al., 2022; Shea, 2008). These measures varied in their approach, assessing spirituality, religiosity, and spiritually injured aspects. Consequently, integrating and comparing findings from these studies presented challenges due to the diversity in measurement approaches.

Two studies (Pereda & Segura, 2021; Pereda et al., 2022) reported that more than half of the participants reported extreme and considerable impacts on their belief in the Church, while nearly half reported similar impacts on their belief in God. However, approximately similar proportions reported none or little impact on their beliefs in both, indicating the need for a more detailed approach, such as qualitative research, to explore the reasons behind these impacts.

Additionally, our review interpreted significant impacts in terms of behavioral actions, such as not attending church services (58.0%), changing religious affiliation (14.0%), and leaving the Church (3.9%) (Dreßing et al., 2019; McLaughlin, 1994), which were still inconsistent in numbers with the two mentioned studies’ findings. In addition to the different sociocultural contexts of each study, the subjectivity of participants’ responses should be considered, especially for the reliability of assessing outcomes of interests; thus, our review was unable to offer statistical estimations of spiritual impacts among survivors of childhood abuse by Catholic clergy.

A variety of mental disorders have been reported, similar to those experienced by survivors of abuse by non-clergy perpetrators (Hailes et al., 2019; Liveri et al., 2023); however, the ramifications of abuse by Catholic clergy go beyond. The abuse unfolded during a vulnerable childhood of survivors who were considerably close to the Catholic Church. The perpetrators were individuals within this sacred institution—the clergy—figures deeply revered and loved by survivors and their families. Disappointing survivors’ trust and profound beliefs through abusive events and unfair or unresponsive actions, both the clergy and the Church are intricately linked to survivors’ faith and beliefs in God, positioned as sources of trust and unconditional love. Consequently, the impact of such abuse extends beyond psychological trauma, as our findings suggest, reaching into the very core of the survivor’s well-being: their spirituality.

Experiences of abuse by trusted and loved ones were identified as betrayal (Mcgraw et al., 2019), leaving several negative consequences (Rachman, 2010) and mistrust, followed by interpersonal difficulties (Green, 1988), as categorized in our first and second themes. However, subtypes of betrayal can be differentiated, including 1) institutional betrayal—harmed by institutions on which survivors rely for safety or survival, and 2) secondary institutional betrayal—survivors' feelings of mistrust toward institutions they are distally connected to, which are not directly involved in their own assault (PettyJohn et al., 2023). In the case of abuse by the clergy, it is more complicated given the involvement of connection not only to the Catholic Church but also to God, which is neither direct nor indirect, depending on personal experiences or ones’ faith before and after abuse.

Due to this complexity, even though we cannot identify all associated factors, it somehow affects the relationship between individuals and God, subsequently introducing life challenges such as diminished sense of life meaning and existentialism (Cranney, 2013). Along with the direct effect of abuse itself, a wide range of negative consequences, as well as suspicious and questions, emerged. It is noteworthy that spiritual impacts alone are not responsible for the adversities and self-doubt that arises, as the abuse itself can be its cause, such as questioning one’s sexual orientations (Roberts et al., 2013). Our study aimed to underscore the intertwined linkage between all domains and spirituality, which is uniquely related to this kind of abuse (Guido, 2008).

It is interesting, but not yet precisely explore, why some survivors decided to remain within the Church. The reason may be due to their own factors or the value that the Church can provide which, at the same time, is irreplaceable and highlights the importance of the Catholic Church.

The themes identified in our studies align with a previous review on the consequences of abuse by religious authorities (Mcgraw et al., 2019). We developed the theme in aspect of difficulties toward others, as described in theme two, and avoided using terms of consequences, concerning multifactorial determinants on one’s spirituality and psychological well-being as well as the lack of evidence from longitudinal quantitative studies have been conducted.

### Limitations

Several limitations should be acknowledged. There were a limited number of studies, particularly those with satisfactory quality. None of the studies included were conducted in Africa and Asia, despite the growing numbers of clergy and Catholic populations in these regions (Segreteria di Stato del Vaticano, 2019). Quantitative studies, particularly those with longitudinal designs capable of demonstrating causal effects, are limited in number. The review included all adult participants from studies on psychological well-being and spirituality, but the sources of participants were not always clearly specified. Several studies were excluded due to language exclusivity and lack of peer-reviewed status, which, for this case, cannot guarantee quality but may introduce publications bias. Additionally, this review primarily focused on the consequences—mostly negative—associated with religious institutions in the context of child abuse. It did not include literature on church- or clergy-led efforts to prevent abuse or to support to survivors and their families. This omission may introduce bias by presenting an incomplete picture of the broader role of religious organizations. Future research should aim to incorporate both protective and positive contributions, including preventive and restorative interventions by faith communities.

Future studies on similar topics are imperative to not only understand the impacts and adulthood status of survivors in different sociocultural contexts but also to estimate the burden and formulate comprehensive policies on this issue. These endeavors would be beneficial for those living under the shadow of abuse and for the Church to continue her role and position as an image of and bridge to our merciful God.

## Conclusion

A broad spectrum of negative psychological conditions and changes in perceptions or beliefs about the Church and God has been observed. However, the causality and sociocultural factors influencing these outcomes remain underexplored. Further research with good methodological quality is needed to understand the complex consequences of clergy abuse within the Catholic Church and to develop supportive policies.

## Supplementary Information

Below is the link to the electronic supplementary material.

Supplementary file1 (DOCX 15 KB)

Supplementary file2 (DOCX 40 KB)
