# Supplementary material for: Spirituality and Psychological Well-Being of Adults with a History of Child Abuse by Catholic Clergy: A Systematic Review of Qualitative and Quantitative Studies
Source: J Relig Health. 2025 Jul 1;64(4):2660–78. doi: 10.1007/s10943-025-02379-3 (PMC12364760; doi:10.1007/s10943-025-02379-3)
Supplement: Supplementary file 1 — Supplementary file1 (DOCX 15 KB) [file 10943_2025_2379_MOESM1_ESM.docx]

**Appendix 1 – Search terms**

Search terms

Spiritual exp spirituality/

(spirit* or religio* or belie* or attitude).ab,ti.

Psychological (psycholog* or emotion or psychiatr* or mental or trauma or anxiety or mood or affect* depress* or quality of li*).ab,ti.

Abuse exp abuse/

(abuse or assault or molestation).ab,ti.

Catholic clergies exp christianity/ and for only EMBASE: exp catholic/

(catholic or clerg* or priest or church).ab,ti
