# Supplementary material for: Spirituality and Psychological Well-Being of Adults with a History of Child Abuse by Catholic Clergy: A Systematic Review of Qualitative and Quantitative Studies
Source: J Relig Health. 2025 Jul 1;64(4):2660–78. doi: 10.1007/s10943-025-02379-3 (PMC12364760; doi:10.1007/s10943-025-02379-3)
Supplement: Supplementary file 2 — Supplementary file2 (DOCX 40 KB) [file 10943_2025_2379_MOESM2_ESM.docx]

**Appendix 2 – the JBI Quality Appraisal Checklist results**

**Study -** McLaughlin, B. R. (1994). Devastated spirituality: The impact of clergy sexual abuse on the survivor’s relationship with god and the church. *Sexual Addiction & Compulsivity*, *1*(2), 145–158.

| **The JBI Critical Appraisal Checklist for analytical cross-sectional study** | | | | |
| --- | --- | --- | --- | --- |
| **Major Components** | **Response options** | | | |
| 1. Were the criteria for inclusion in the sample clearly defined? | **Yes** | No | Unclear | Not applicable |
| 2. Were the study subjects and the setting described in detail? | **Yes** | No | Unclear | Not applicable |
| 3. Was the exposure measured in a valid and reliable way? | Yes | No | **Unclear** | Not applicable |
| 4. Were objective, standard criteria used for measurement of the condition? | Yes | No | **Unclear** | Not applicable |
| 5. Were confounding factors identified? | Yes | No | **Unclear** | Not applicable |
| 6. Were strategies to deal with confounding factors stated? | Yes | No | **Unclear** | Not applicable |
| 7. Were the outcomes measured in a valid and reliable way? | **Yes** | No | Unclear | Not applicable |
| 8. Was appropriate statistical analysis used? | **Yes** | No | Unclear | Not applicable |
| **Overall appraisal:** Include [X] Exclude [ ] Seek further info [ ] | | | | |

**Study -** Shea, D. J. (2008). Effects of Sexual Abuse by Catholic Priests on Adults Victimized as Children. *Sexual Addiction & Compulsivity*, *15*(3), 250–268.

| **The JBI Critical Appraisal Checklist for analytical cross-sectional study** | | | | |
| --- | --- | --- | --- | --- |
| **Major Components** | **Response options** | | | |
| 1. Were the criteria for inclusion in the sample clearly defined? | **Yes** | No | Unclear | Not applicable |
| 2. Were the study subjects and the setting described in detail? | Yes | No | **Unclear** | Not applicable |
| 3. Was the exposure measured in a valid and reliable way? | **Yes** | No | Unclear | Not applicable |
| 4. Were objective, standard criteria used for measurement of the condition? | **Yes** | No | Unclear | Not applicable |
| 5. Were confounding factors identified? | Yes | No | **Unclear** | Not applicable |
| 6. Were strategies to deal with confounding factors stated? | Yes | No | **Unclear** | Not applicable |
| 7. Were the outcomes measured in a valid and reliable way? | **Yes** | No | Unclear | Not applicable |
| 8. Was appropriate statistical analysis used? | **Yes** | No | Unclear | Not applicable |
| **Overall appraisal:** Include [X] Exclude [ ] Seek further info [ ] | | | | |

**Study -** Lueger-Schuster, B., Kantor, V., Weindl, D., Knefel, M., Moy, Y., Butollo, A., Jagsch, R., & Glück, T. (2014). Institutional abuse of children in the Austrian Catholic Church: Types of abuse and impact on adult survivors’ current mental health. *Child Abuse & Neglect*, *38*(1), 52–64.

| **The JBI Critical Appraisal Checklist for analytical cross-sectional study** | | | | |
| --- | --- | --- | --- | --- |
| **Major Components** | **Response options** | | | |
| 1. Were the criteria for inclusion in the sample clearly defined? | **Yes** | No | Unclear | Not applicable |
| 2. Were the study subjects and the setting described in detail? | **Yes** | No | Unclear | Not applicable |
| 3. Was the exposure measured in a valid and reliable way? | Yes | No | **Unclear** | Not applicable |
| 4. Were objective, standard criteria used for measurement of the condition? | **Yes** | No | Unclear | Not applicable |
| 5. Were confounding factors identified? | Yes | No | **Unclear** | Not applicable |
| 6. Were strategies to deal with confounding factors stated? | Yes | No | **Unclear** | Not applicable |
| 7. Were the outcomes measured in a valid and reliable way? | **Yes** | No | Unclear | Not applicable |
| 8. Was appropriate statistical analysis used? | **Yes** | No | Unclear | Not applicable |
| **Overall appraisal:** Include [X] Exclude [ ] Seek further info [ ] | | | | |

**Study -** Lueger-Schuster, B., Weindl, D., Kantor, V., Knefel, M., Glück, T., Moy, Y., Butollo, A., & Jagsch, R. (2014). Resilience and mental health in adult survivors of child abuse associated with the institution of the Austrian Catholic Church. *Journal of Traumatic Stress*, *27*(5), 568–575.

| **The JBI Critical Appraisal Checklist for analytical cross-sectional study** | | | | |
| --- | --- | --- | --- | --- |
| **Major Components** | **Response options** | | | |
| 1. Were the criteria for inclusion in the sample clearly defined? | **Yes** | No | Unclear | Not applicable |
| 2. Were the study subjects and the setting described in detail? | **Yes** | No | Unclear | Not applicable |
| 3. Was the exposure measured in a valid and reliable way? | Yes | No | **Unclear** | Not applicable |
| 4. Were objective, standard criteria used for measurement of the condition? | **Yes** | No | Unclear | Not applicable |
| 5. Were confounding factors identified? | Yes | No | **Unclear** | Not applicable |
| 6. Were strategies to deal with confounding factors stated? | Yes | No | **Unclear** | Not applicable |
| 7. Were the outcomes measured in a valid and reliable way? | **Yes** | No | Unclear | Not applicable |
| 8. Was appropriate statistical analysis used? | **Yes** | No | Unclear | Not applicable |
| **Overall appraisal:** Include [X] Exclude [ ] Seek further info [ ] | | | | |

**Study -** Spröber, N., Schneider, T., Rassenhofer, M., Seitz, A., Liebhardt, H., König, L., & Fegert, J. M. (2014). Child sexual abuse in religiously affiliated and secular institutions: A retrospective descriptive analysis of data provided by victims in a government-sponsored reappraisal program in Germany. *BMC Public Health*, *14*, 282.

| **The JBI Critical Appraisal Checklist for analytical cross-sectional study** | | | | |
| --- | --- | --- | --- | --- |
| **Major Components** | **Response options** | | | |
| 1. Were the criteria for inclusion in the sample clearly defined? | **Yes** | No | Unclear | Not applicable |
| 2. Were the study subjects and the setting described in detail? | **Yes** | No | Unclear | Not applicable |
| 3. Was the exposure measured in a valid and reliable way? | Yes | No | **Unclear** | Not applicable |
| 4. Were objective, standard criteria used for measurement of the condition? | Yes | No | **Unclear** | Not applicable |
| 5. Were confounding factors identified? | Yes | No | **Unclear** | Not applicable |
| 6. Were strategies to deal with confounding factors stated? | Yes | No | **Unclear** | Not applicable |
| 7. Were the outcomes measured in a valid and reliable way? | Yes | No | **Unclear** | Not applicable |
| 8. Was appropriate statistical analysis used? | **Yes** | No | Unclear | Not applicable |
| **Overall appraisal:** Include [X] Exclude [ ] Seek further info [ ] | | | | |

**Study -** Pereda, N., & Segura, A. (2021). Child sexual abuse within the Roman Catholic Church in Spain: A descriptive study of abuse characteristics, victims’ faith, and spirituality. *Psychology of Violence*, *11*(5), 488–496

| **The JBI Critical Appraisal Checklist for analytical cross-sectional study** | | | | |
| --- | --- | --- | --- | --- |
| **Major Components** | **Response options** | | | |
| 1. Were the criteria for inclusion in the sample clearly defined? | **Yes** | No | Unclear | Not applicable |
| 2. Were the study subjects and the setting described in detail? | **Yes** | No | Unclear | Not applicable |
| 3. Was the exposure measured in a valid and reliable way? | Yes | No | **Unclear** | Not applicable |
| 4. Were objective, standard criteria used for measurement of the condition? | Yes | No | **Unclear** | Not applicable |
| 5. Were confounding factors identified? | Yes | No | **Unclear** | Not applicable |
| 6. Were strategies to deal with confounding factors stated? | Yes | No | **Unclear** | Not applicable |
| 7. Were the outcomes measured in a valid and reliable way? | **Yes** | No | Unclear | Not applicable |
| 8. Was appropriate statistical analysis used? | **Yes** | No | Unclear | Not applicable |
| **Overall appraisal:** Include [X] Exclude [ ] Seek further info [ ] | | | | |

**Study -** Pereda, N., Contreras Taibo, L., Segura, A., & Maffioletti Celedón, F. (2022). An Exploratory Study on Mental Health, Social Problems and Spiritual Damage in Victims of Child Sexual Abuse by Catholic Clergy and Other Perpetrators. *Journal of Child Sexual Abuse*, *31*(4), 393–411.

| **The JBI Critical Appraisal Checklist for analytical cross-sectional study** | | | | |
| --- | --- | --- | --- | --- |
| **Major Components** | **Response options** | | | |
| 1. Were the criteria for inclusion in the sample clearly defined? | **Yes** | No | Unclear | Not applicable |
| 2. Were the study subjects and the setting described in detail? | Yes | No | **Unclear** | Not applicable |
| 3. Was the exposure measured in a valid and reliable way? | Yes | No | **Unclear** | Not applicable |
| 4. Were objective, standard criteria used for measurement of the condition? | Yes | No | **Unclear** | Not applicable |
| 5. Were confounding factors identified? | **Yes** | No | Unclear | Not applicable |
| 6. Were strategies to deal with confounding factors stated? | **Yes** | No | Unclear | Not applicable |
| 7. Were the outcomes measured in a valid and reliable way? | Yes | No | **Unclear** | Not applicable |
| 8. Was appropriate statistical analysis used? | **Yes** | No | Unclear | Not applicable |
| **Overall appraisal:** Include [X] Exclude [ ] Seek further info [ ] | | | | |

**Study -** Dreßing, H., Dölling, D., Hermann, D., Kruse, A., Schmitt, E., Bannenberg, B., Hoell, A., Voss, E., & Salize, H. J. (2019). Sexual Abuse at the Hands of Catholic Clergy. *Deutsches Arzteblatt International*, *116*(22), 389–396.

| **The JBI Critical Appraisal Checklist for prevalence study** | | | | |
| --- | --- | --- | --- | --- |
| **Major Components** | **Response options** | | | |
| 1. Was the sample frame appropriate to address the target population? | **Yes** | No | Unclear | Not applicable |
| 2. Were study participants sampled in an appropriate way? | **Yes** | No | Unclear | Not applicable |
| 3. Was the sample size adequate? | **Yes** | No | Unclear | Not applicable |
| 4. Were the study subjects and the setting described in detail? | **Yes** | No | Unclear | Not applicable |
| 5. Was the data analysis conducted with sufficient coverage of the identified sample? | Yes | No | Unclear | **Not applicable** |
| 6. Were valid methods used for the identification of the condition? | Yes | No | **Unclear** | Not applicable |
| 7. Was the condition measured in a standard, reliable way for all participants? | **Yes** | No | Unclear | Not applicable |
| 8. Was there appropriate statistical analysis? | Yes | No | Unclear | **Not applicable** |
| 9. Was the response rate adequate, and if not, was the low response rate managed appropriately? | **Yes** | No | Unclear | Not applicable |
| **Overall appraisal:** Include [X] Exclude [ ] Seek further info [ ] | | | | |

**Study -** Mart, E. G. (2004). Victims of Abuse by Priests: Some Preliminary Observations. *Pastoral Psychology*, *52*(6), 465–472.

| **The JBI Critical Appraisal Checklist for qualitative study** | | | | |
| --- | --- | --- | --- | --- |
| **Major Components** | **Response options** | | | |
| 1. Is there congruity between the stated philosophical perspective and the research   methodology? | **Yes** | No | Unclear | Not applicable |
| 2. Is there congruity between the research methodology and the research question or objectives? | **Yes** | No | Unclear | Not applicable |
| 3. Is there congruity between the research methodology and the methods used to collect data? | **Yes** | No | Unclear | Not applicable |
| 4. Is there congruity between the research methodology and the representation and analysis of   data? | Yes | No | **Unclear** | Not applicable |
| 5. Is there congruity between the research methodology and the interpretation of results? | **Yes** | No | Unclear | Not applicable |
| 6. Is there a statement locating the researcher culturally or theoretically? | **Yes** | No | Unclear | Not applicable |
| 7. Is the influence of the researcher on the research, and vice-versa, addressed? | Yes | No | **Unclear** | Not applicable |
| 8. Are participants, and their voices, adequately represented? | Yes | No | **Unclear** | Not applicable |
| 9. Is the research ethical according to current criteria or is there evidence of ethical approval by an   appropriate body? | Yes | No | **Unclear** | Not applicable |
| 10. Do the conclusions drawn in the research report flow from the analysis, or interpretation, of   the data? | **Yes** | No | Unclear | Not applicable |
| **Overall appraisal:** Include [X] Exclude [ ] Seek further info [ ] | | | | |

**Study -** Isely, P. J., Isely, P., Freiburger, J., & McMackin, R. (2008). In their own voices: A qualitative study of men abused as children by catholic clergy. *Journal of Child Sexual Abuse*, *17*(3–4), 201–215.

| **The JBI Critical Appraisal Checklist for qualitative study** | | | | |
| --- | --- | --- | --- | --- |
| **Major Components** | **Response options** | | | |
| 1. Is there congruity between the stated philosophical perspective and the research   methodology? | **Yes** | No | Unclear | Not applicable |
| 2. Is there congruity between the research methodology and the research question or objectives? | **Yes** | No | Unclear | Not applicable |
| 3. Is there congruity between the research methodology and the methods used to collect data? | **Yes** | No | Unclear | Not applicable |
| 4. Is there congruity between the research methodology and the representation and analysis of   data? | **Yes** | No | Unclear | Not applicable |
| 5. Is there congruity between the research methodology and the interpretation of results? | **Yes** | No | Unclear | Not applicable |
| 6. Is there a statement locating the researcher culturally or theoretically? | Yes | No | **Unclear** | Not applicable |
| 7. Is the influence of the researcher on the research, and vice-versa, addressed? | Yes | No | **Unclear** | Not applicable |
| 8. Are participants, and their voices, adequately represented? | **Yes** | No | Unclear | Not applicable |
| 9. Is the research ethical according to current criteria or is there evidence of ethical approval by an   appropriate body? | Yes | No | **Unclear** | Not applicable |
| 10. Do the conclusions drawn in the research report flow from the analysis, or interpretation, of   the data? | **Yes** | No | Unclear | Not applicable |
| **Overall appraisal:** Include [X] Exclude [ ] Seek further info [ ] | | | | |

**Study -** Farrell, D. P. (2009). Sexual abuse perpetrated by Roman Catholic priests and religious. *Mental Health, Religion & Culture*, *12*(1), 39–53.

| **The JBI Critical Appraisal Checklist for qualitative study** | | | | |
| --- | --- | --- | --- | --- |
| **Major Components** | **Response options** | | | |
| 1. Is there congruity between the stated philosophical perspective and the research   methodology? | **Yes** | No | Unclear | Not applicable |
| 2. Is there congruity between the research methodology and the research question or objectives? | **Yes** | No | Unclear | Not applicable |
| 3. Is there congruity between the research methodology and the methods used to collect data? | **Yes** | No | Unclear | Not applicable |
| 4. Is there congruity between the research methodology and the representation and analysis of   data? | **Yes** | No | Unclear | Not applicable |
| 5. Is there congruity between the research methodology and the interpretation of results? | **Yes** | No | Unclear | Not applicable |
| 6. Is there a statement locating the researcher culturally or theoretically? | Yes | **No** | Unclear | Not applicable |
| 7. Is the influence of the researcher on the research, and vice-versa, addressed? | Yes | No | **Unclear** | Not applicable |
| 8. Are participants, and their voices, adequately represented? | **Yes** | No | Unclear | Not applicable |
| 9. Is the research ethical according to current criteria or is there evidence of ethical approval by an   appropriate body? | **Yes** | No | Unclear | Not applicable |
| 10. Do the conclusions drawn in the research report flow from the analysis, or interpretation, of   the data? | **Yes** | No | Unclear | Not applicable |
| **Overall appraisal:** Include [X] Exclude [ ] Seek further info [ ] | | | | |

**Study -** Easton, S. D., Leone-Sheehan, D. M., & O’Leary, P. J. (2019). “I Will Never Know the Person Who I Could Have Become”: Perceived Changes in Self-Identity Among Adult Survivors of Clergy-Perpetrated Sexual Abuse. *Journal of Interpersonal Violence*, *34*(6), 1139–1162.

| **The JBI Critical Appraisal Checklist for qualitative study** | | | | |
| --- | --- | --- | --- | --- |
| **Major Components** | **Response options** | | | |
| 1. Is there congruity between the stated philosophical perspective and the research   methodology? | **Yes** | No | Unclear | Not applicable |
| 2. Is there congruity between the research methodology and the research question or objectives? | **Yes** | No | Unclear | Not applicable |
| 3. Is there congruity between the research methodology and the methods used to collect data? | **Yes** | No | Unclear | Not applicable |
| 4. Is there congruity between the research methodology and the representation and analysis of   data? | **Yes** | No | Unclear | Not applicable |
| 5. Is there congruity between the research methodology and the interpretation of results? | **Yes** | No | Unclear | Not applicable |
| 6. Is there a statement locating the researcher culturally or theoretically? | Yes | No | **Unclear** | Not applicable |
| 7. Is the influence of the researcher on the research, and vice-versa, addressed? | Yes | No | **Unclear** | Not applicable |
| 8. Are participants, and their voices, adequately represented? | **Yes** | No | Unclear | Not applicable |
| 9. Is the research ethical according to current criteria or is there evidence of ethical approval by an   appropriate body? | **Yes** | No | Unclear | Not applicable |
| 10. Do the conclusions drawn in the research report flow from the analysis, or interpretation, of   the data? | **Yes** | No | Unclear | Not applicable |
| **Overall appraisal:** Include [X] Exclude [ ] Seek further info [ ] | | | | |

**Study -** Prusak, J., & Schab, A. (2022). Spiritual trauma as a manifestation of religious and spiritual struggles in female victims of sexual abuse in adolescence or young adulthood in the Catholic Church in Poland. *Archive for the Psychology of Religion*, *44*(1), 40–65.

| **The JBI Critical Appraisal Checklist for qualitative study** | | | | |
| --- | --- | --- | --- | --- |
| **Major Components** | **Response options** | | | |
| 1. Is there congruity between the stated philosophical perspective and the research   methodology? | **Yes** | No | Unclear | Not applicable |
| 2. Is there congruity between the research methodology and the research question or objectives? | **Yes** | No | Unclear | Not applicable |
| 3. Is there congruity between the research methodology and the methods used to collect data? | **Yes** | No | Unclear | Not applicable |
| 4. Is there congruity between the research methodology and the representation and analysis of   data? | **Yes** | No | Unclear | Not applicable |
| 5. Is there congruity between the research methodology and the interpretation of results? | **Yes** | No | Unclear | Not applicable |
| 6. Is there a statement locating the researcher culturally or theoretically? | **Yes** | No | Unclear | Not applicable |
| 7. Is the influence of the researcher on the research, and vice-versa, addressed? | Yes | No | **Unclear** | Not applicable |
| 8. Are participants, and their voices, adequately represented? | **Yes** | No | Unclear | Not applicable |
| 9. Is the research ethical according to current criteria or is there evidence of ethical approval by an   appropriate body? | **Yes** | No | Unclear | Not applicable |
| 10. Do the conclusions drawn in the research report flow from the analysis, or interpretation, of   the data? | **Yes** | No | Unclear | Not applicable |
| **Overall appraisal:** Include [X] Exclude [ ] Seek further info [ ] | | | | |
